# The MHV68 M2 Protein Drives IL-10 Dependent B Cell Proliferation and Differentiation

**DOI:** 10.1371/journal.ppat.1000039

**Published:** 2008-04-04

**Authors:** Andrea M. Siegel, Jeremy H. Herskowitz, Samuel H. Speck

**Affiliations:** Emory Vaccine Center and Department of Microbiology & Immunology, Emory University School of Medicine, Atlanta, Georgia, United States of America; University of Wisconsin-Madison, United States of America

## Abstract

Murine gammaherpesvirus 68 (MHV68) establishes long-term latency in memory B cells similar to the human gammaherpesvirus Epstein Barr Virus (EBV). EBV encodes an interleukin-10 (IL-10) homolog and modulates cellular IL-10 expression; however, the role of IL-10 in the establishment and/or maintenance of chronic EBV infection remains unclear. Notably, MHV68 does not encode an IL-10 homolog, but virus infection has been shown to result in elevated serum IL-10 levels in wild-type mice, and IL-10 deficiency results in decreased establishment of virus latency. Here we show that a unique MHV68 latency-associated gene product, the M2 protein, is required for the elevated serum IL-10 levels observed at 2 weeks post-infection. Furthermore, M2 protein expression in primary murine B cells drives high level IL-10 expression along with increased secretion of IL-2, IL-6, and MIP-1α. M2 expression was also shown to significantly augment LPS driven survival and proliferation of primary murine B cells. The latter was dependent on IL-10 expression as demonstrated by the failure of IL10^−/−^ B cells to proliferate in response to M2 protein expression and rescue of M2-associated proliferation by addition of recombinant murine IL-10. M2 protein expression in primary B cells also led to upregulated surface expression of the high affinity IL-2 receptor (CD25) and the activation marker GL7, along with down-regulated surface expression of B220, MHC II, and sIgD. The cells retained CD19 and sIgG expression, suggesting differentiation to a pre-plasma memory B cell phenotype. These observations are consistent with previous analyses of M2-null MHV68 mutants that have suggested a role for the M2 protein in expansion and differentiation of MHV68 latently infected B cells—perhaps facilitating the establishment of virus latency in memory B cells. Thus, while the M2 protein is unique to MHV68, analysis of M2 function has revealed an important role for IL-10 in MHV68 pathogenesis—identifying a strategy that appears to be conserved between at least EBV and MHV68.

## Author Summary

Gammaherpesviruses are able to maintain life-long, quiescent infections (latency) in lymphocytes characterized by intermittent production of infectious progeny virus (reactivation). The murine gammaherpesvirus 68 (MHV68) has extensive genetic and phenotypic similarities to the human gammaherpesviruses Epstein-Barr virus (EBV) and Kaposi's sarcoma associated herpesvirus (KSHV). Similar to EBV pathogenesis, MHV68 establishes long-term latency in memory B cells. A unique MHV68 protein designated M2 is known to play an important role in both establishment of latency and reactivation from latency. Efficient transition of MHV68 latency to the memory B cell population is hindered in the absence of M2, leading to the hypothesis that M2 may be involved in MHV68-driven B cell differentiation. In this study we show that M2 expression enhanced primary murine B cell survival, proliferation, and differentiation in culture. M2 expressing B cells secreted of high levels of IL-10 that is necessary for the observed expansion of the M2-expressing B cell population *in vitro.* Mice infected with a M2-null MHV68 mutant had a significant decrease in serum IL-10, and this correlated with an increased frequency of MHV68-specific CD8^+^ T cells in these animals. Thus, M2 manipulation of IL-10 signaling appears to both drive expansion of the major latency reservoir (B cells), as well as dampen the immune response to the virus facilitating both viral latency and reactivation.

## Introduction

Herpesviruses establish life-long, latent infections characterized by episodic virus reactivation and subsequent virus shedding. Chronic infections with the lymphotropic gammaherpesviruses are associated with a variety of lymphomas and carcinomas which in humans includes Burkitt's lymphoma, nasopharyngeal carcinoma, Hodgkin's disease and Kaposi's sarcoma. The narrow host range of the gammaherpesviruses that infect humans, Epstein-Barr Virus (EBV) and Kaposi's sarcoma-associated herpesvirus (KSHV), has severely hindered detailed pathogenesis studies. Murine gammaherpesvirus 68 (MHV68; also known as γHV68 and murine herpesvirus 4) shares extensive genetic homology and biological similarity with both EBV and KSHV and is a natural pathogen of wild murid rodents. As such, MHV68 infection of inbred strains of mice has gained favor as a small animal model in which to evaluate viral and host determinants of gammaherpesvirus pathogenesis *in vivo*.

Upon intranasal infection, MHV68 infection results in acute viremia in the lung that is later resolved into a latent infection of B cells, dendritic cells, and macrophages [1]. B cells are necessary for trafficking of virally infected cells to the spleen, leading to the establishment of splenic latency [2],[3]. The CD8^+^ T cell response is critical for control of lytic infection in the lung as well as establishment of latent viral load in the spleen [4]. MHV68 infection results in a CD4^+^ T cell-dependent expansion of splenic B cells and both virus-specific and non-specific hypergammaglobulinemia [5],[6]. Similar to EBV pathogenesis, memory B cells are the primary long-term reservoir of latent MHV68 in mice [7],[8],[9].

All herpesviruses manipulate the host's immune system to establish and maintain a long-term, latent infection, and many of these immunomodulatory mechanisms are conserved among the members of the gammaherpesvirus family. Both KSHV and MHV68 encode proteins, K3 and mK3, respectively, that downregulate MHC I [10]. MHV68 also encodes a viral bcl-2 homolog, a viral cyclin, and a chemokine-binding protein, M3 [11],[12],[13]. The EBV proteins LMP1 and LMP2a mimic CD40 and tonic BCR signals, respectively, to manipulate B cell development and are believed to enable the virus to gain access to the memory B cell compartment independent of antigenic stimulation of the host B cell [14],[15]. KSHV encodes both a viral IL-6 and a viral MIP-1α ortholog, while EBV encodes a viral IL-10 homolog, BCRF1 (or vIL-10) [16],[17].

Interleukin-10 (IL-10) was first noted as a cytokine synthesis inhibitory factor (CSIF) that is secreted by T_H_2 cells and suppresses the activity of T_H_1 cells [18]. IL-10 enhances murine B cell viability and can activate human B cell proliferation and class switching in culture [17],[19]. In addition, IL-10 suppresses T_H_1 responses through modulation of macrophage function by downregulation of MHC II and costimulatory molecules as well as inhibition of cytokine production and macrophage effector functions [20],[21]. Dendritic cells exposed to IL-10 do not down-regulate costimulatory molecules but do secrete lower levels of IL-12, impairing their ability to induce a T_H_1 response [22].

The M2 protein, unique to MHV68, has been shown to play a critical role in both the establishment of latency as well as reactivation from latency [23],[24],[25]. A M2-null strain of MHV68 (MHV68/M2.Stop) replicates with wild-type efficiency in mice following intranasal inoculation but exhibits a dose-dependent defect in the establishment of latency at day 16 post-infection [23],[24]. Under conditions in which the MHV68/M2.Stop mutant can efficiently establish a latent infection (high dose intranasal inoculation or low dose intraperitoneal inoculation), the M2-null virus exhibits a profound reactivation defect, revealing dual roles for the M2 protein in the viral life-cycle [23],[24]. Additionally, efficient transition of latently-infected B cells from the germinal center reaction to the memory B cell reservoir appears to be stalled in the absence of M2, suggesting M2 may manipulate B cell signaling or differentiation to facilitate establishment of long-term latency in the memory B cell pool [24],[26]. Numerous candidate SH3 binding motifs throughout M2 suggest the protein may function as a molecular scaffold that may modulate specific cellular signal transduction pathways. Consistent with this hypothesis, M2 has been shown to interact with a number of cellular proteins in vitro. M2 co-immunoprecipitates with Vav1 in S11 B cells, a MHV68 latently infected cell line, and M2 and Vav1 overexpression in A20 B cells leads to Vav1 phosphorylation, trimerization with Fyn, and downstream activation of Rac1 [27]. In fibroblast cultures, M2 interacts with DDB1/COP9/cullin repair complex and ATM to suppress DNA-damage induced apoptosis [28]. In addition, M2 can suppress STAT1/2 expression, leading to inhibition of the interferon response [29]. However, to date the impact of M2 expression in primary murine B cells has not been reported.

Here we show that one function of the M2 protein is to induce expression of IL-10 in primary B cells, demonstrating a common immunomodulatory strategy utilized by those gammaherpesviruses encoding a viral IL-10 homolog and MHV68.

## Results

### MH68 M2 protein augments LPS-driven B cell proliferation

**Figure 1:**
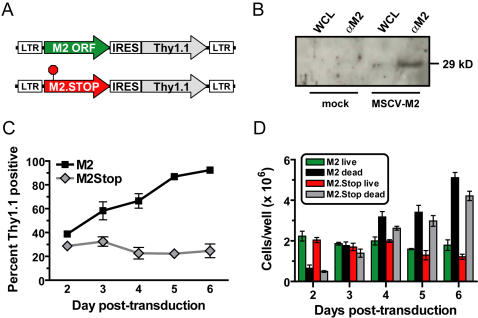
M2 expression in primary B cells leads to expansion of the tranduced population. (A) The M2 open reading frame and M2 ORF with a stop codon at amino acid number 13 (M2.Stop) were cloned into the MSCV-IRES-Thy1.1 vector. (B) Expression of M2 protein in primary murine B cells four days post-transduction. B cells were lysed in CHAPS-BOG and immunoprecipitated with chicken anti-M2 IgY and blotted with rabbit anti-M2 antisera. (C) M2-transduced B cells expand in culture over time. B cells were purified by negative selection MACS separation, and B cell cultures were between 94–97% pure (gated on CD19^+^). Triplicate B cell cultures were transduced with M2 or M2.Stop retroviruses and allowed to rest for 48 hours before analysis. Cells were stained with anti-Thy1.1 and analyzed daily for expression. Data representative of more than three independent experiments. (D) Absolute numbers of cells per well are unchanged over time. Trypan exclusion was used to count absolute numbers of live and dead cells in triplicate wells per day. Data representative of at least two independent experiments.

The MHV68 M2 protein has been shown to be critical for both establishment and reactivation from B cell latency. M2 has no known homologous proteins, viral or cellular, and contains numerous SH3 binding motifs through which it can potentially manipulate B cell biology. Proliferating B cells harbor the majority of latent MHV68 genomes, and splenic B cell activation is associated with MHV68 infection at the onset of latency [30],[31]. We asked whether expression of M2 in primary murine B cells in vitro altered proliferation or activation. B cells were purified by negative selection from mouse splenocytes, stimulated overnight with LPS, and transduced with either an M2 protein expressing recombinant murine stem cell virus (MSCV) retrovirus, MSCV-M2-IRES-Thy1.1, or a control retrovirus, MSCV-M2.Stop-IRES-Thy1.1, which harbors a translation termination codon near the 5′ end of the M2 open reading frame at amino acid 13 (Figure 1A). LPS stimulation is necessary for efficient retroviral transduction in this system because MSCV infection requires the cells to be in cycle [32]. The presence of an IRES-Thy1.1 cassette readily allowed retroviral transduction efficiency to be monitored by flow cytometry for surface expression of Thy1.1. Notably, expression of the M2 protein from the retroviral construct could be detected as demonstrated by immunoprecipitation and immunoblotting of whole cell lysates harvested from primary B cells transduced with MSCV-M2-IRES-Thy1.1 at four days post-transduction (Figure 1B). It should be noted that detection of M2 expression in the transduced primary murine B cells required immunoprecipitation with a chicken anti-M2 antisera raised against two M2 peptides, followed by immunoblotting with a rabbit polyclonal antiserum raised against a bacterially expression recombinant M2 protein. In contrast, M2 expression in the MHV68 latently infected B lymphoma cell line S11 can be detected by immunoblotting S11 lysates with the rabbit polyclonal anti-M2 antiserum (data not shown). Thus, it does not appear that M2 is “over-expressed” in transduced primary murine B cells.

Two days post-transduction, there were similar frequencies of transduced, Thy1.1^+^ B cells in the control and M2-transduced B cell cultures. However, by 5–6 days post-transduction, nearly 100% of the B cell culture transduced with MSCV-M2-IRES-Thy1.1 was Thy1.1^+^ as compared to ca. 20% of the culture transduced with the control vector, MSCV-M2.Stop-IRES-Thy1.1 (Figure 1C). Notably, the dominance of M2-expressing, Thy1.1^+^ B cells in the M2-transduced cultures was observed repeatedly. The increase in the percentage of Thy1.1^+^ cells in the M2-transduced culture was gradual, and it did not correspond to an increase in overall cell number in the cultures or a decrease in cell death (Figure 1D). The latter result suggests that in a mixed culture (M2 expressing and non-expressing cells), the non-transduced primary B cells are actively selected against. This could either be due to the secretion of a “toxic” factor by the M2 expressing cells or competition for a limiting factor necessary for cell survival.

**Figure 2:**
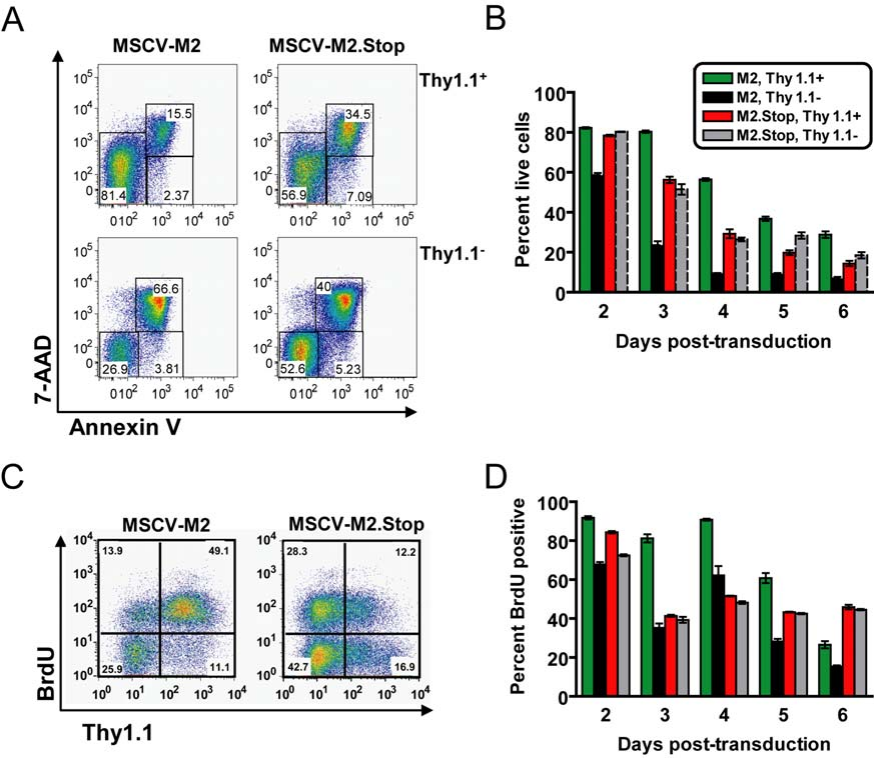
M2 expression leads to enhanced survival and B cell proliferation. (A and B) M2-transduced cells have enhanced survival versus the untransduced cells in culture as well as M2.Stop-transduced cells. Triplicate cultures were stained with Thy1.1-PE, 7-AAD, and AnnexinV-PacificBlue and analyzed on a LSRII cytometer. (A) Representative data from 3 days post-transduction excluding obvious debris in the culture, gated on Thy1.1 expression. (B) The percentage of live (AnnexinV^−^ 7-AAD^−^) was determined from 2–6 days post-retroviral transduction with M2 or M2.Stop in transduced (Thy1.1^+^) and untransduced (Thy1.1^−^) populations. Data is representative of two independent time-course experiments. (C and D) M2-transduced B cells had enhanced proliferation early post-transduction. Triplicate B cell cultures were pulsed with 10 µM BrdU for 24 hours and stained with Thy1.1-PE and BrdU-APC and analyzed on a FACScalibur. (C) Representative data from day 3 post-transduction shows M2-enhanced proliferation of the transduced (Thy1.1^+^) population, gated on viable cells. (D) Percent of transduced, proliferating cells over time demonstrates enhanced proliferation in B cells transduced with M2 at days 3 and 4 post-transduction. Proliferation of the untransduced populations were similar over the time-course. Statistical significance of the flow cytometry data was determined by two-tailed, unpaired Student's T test with a confidence level of 95%.

Upon observing the M2-transduced cells dominating the culture, we asked whether M2 was influencing B cell survival, proliferation, or both. To directly assess B cell survival in M2-transduced and control retrovirus cultures (M2.Stop), cells were stained with anti-Thy1.1, Annexin V, and 7-AAD and analyzed by flow cytometry. In contrast to the results obtained by trypan blue exclusion which measured the live/dead ratio in the entire culture (Figure 1D), flow cytometry of the transduced and untransduced populations within the culture revealed a survival advantage of the M2-transduced B cells. At day 2 post-transduction, 20% more of the M2-transduced B cells were alive (AnnexinV^−^ 7-AAD^−^) than the untransduced cells in the same culture (Figure 2B). At day 3 post-transduction, there was a four-fold higher frequency of live cells in the M2-transduced population as compared to the untransduced cells in culture (Figure 2, panels A & B). M2-transduced cells continued to survive better than the untransduced cells in the population, despite an equal frequency of cells entering apoptosis (data not shown).

At day 2 post-transduction, the M2-transduced cells have equal frequencies of live cells as the control M2.Stop retrovirus transduced cells (Figure 2B). Analysis at day 3 post-transduction revealed a 20% increase in the frequency of live cells in the M2-transduced population as compared to the cells transduced with the control retrovirus (Figure 2B). This trend continued until the end of the time-course, with a higher frequency of live cells found in the M2-transduced population versus the M2.Stop retrovirus control (Figure 2B). The increased frequency of live cells in the M2-transduced population versus both the untransduced cells within the culture as well as the control retrovirus transduced population reveals a pro-survival effect of M2 protein expression in B cells.

We next addressed whether M2 protein expression altered proliferation in the B cell cultures thereby contributing to the expansion of transduced cells. To directly assess B cell proliferation in M2-transduced and control retrovirus cultures, cells were pulsed with bromodeoxyuradine (BrdU) for 24 hours at different time points post-transduction. Cells were surface stained for Thy1.1 and proliferation was measured by intracellular staining for incorporation of BrdU. The time course analyses revealed that B cells transduced with either the M2 or control retrovirus exhibited equivalent frequencies (84–90%) of proliferating cells 2 days post-transduction (Figure 2D). However, 80–90% of M2-transduced B cells continued to proliferate 3 and 4 days post-transduction as compared to 40–50% of the cells transduced with the control retrovirus (Figures 2, panel C & D). By 5–6 days post-transduction there was a significant drop in the proliferation of M2-transduced B cells (Figure 2D). These results indicate that M2 protein expression is able to transiently augment murine B cell proliferation. Thus, these analyses indicated that the M2 protein contributes to both enhanced B cell survival as well as promoting continued B cell proliferation post-LPS stimulation – which together leads to dominance of M2-transduced B cells in the primary murine B cell cultures over the time-course analyzed.

### M2 protein expression leads to B cell differentiation

**Figure 3:**
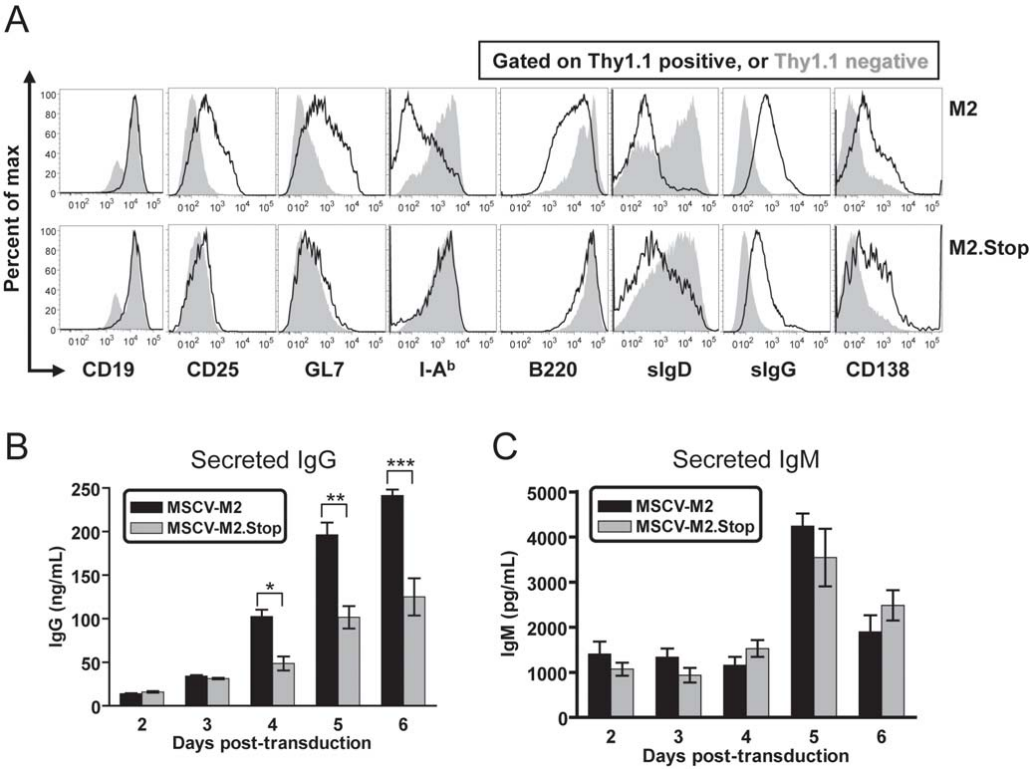
M2 expressing cells have an activated, pre-plasma memory phenotype. (A) M2-transduced cells were CD19^+^, CD25^high^, GL7^high^, B220^low^, I-A^b low^, sIgD^−^, sIgG^+^, and CD138^low^ when compared to untransduced cells within the culture. Representative flow cytometry histograms at day 4 post-transduction. The black line open histograms reflect staining of the transduced (Thy1.1^+^) B cell population, while the filled gray histograms reflect staining of the untransduced (Thy1.1^−^) population. The top panels depict data from M2-transduced cultures; bottom panels depict data from M2.Stop-transduced cells. Data is representative of three samples per time-point with at least three independent experiments per stain. (B and C) ELISA quantitation of the levels of IgM and IgG in supernatants of M2 and M2.Stop transduced B cell cultures. Three samples were analyzed per time point, and the data shown is representative of three independent experiments. Significance of differences in IgG secretion was determined by two-tailed, unpaired Student's T test with a confidence level of 95%. * p = 0.0182, ** p = 0.0352, *** p = 0.0118.

The transition from the germinal center B cell population to the long-lived memory B cell compartment is critical for establishment of MHV68 latency [8],[9]. Latent genomes in mice infected with M2-deficent MHV68 accumulate in the germinal center compartment late in infection, leading to the hypothesis that M2 is capable of manipulating B cell differentiation [26]. To determine whether M2-transduction leads to differentiation of B cells, surface expression of B cell differentiation markers was analyzed by flow cytometry. At four days post-transduction, B cells expressing M2 were CD19^+^, CD25^high^, GL7^high^, B220^low^, I-A^b low^, surface IgD^−^ (sIgD), sIgG^+^, and CD138^low^ when compared to untransduced cells within the culture (Figure 3A). Strikingly, M2-transduced cells expressed higher levels of CD25 as compared to cells transduced with the control retrovirus, although the MFI of CD25 was similar between the two populations (Figure 3A). Both transduced populations (M2 and M2.Stop) became surface IgG positive, likely due to LPS stimulation coupled with retrovirus infection selecting for the LPS-driven proliferating B cell population. However, the M2 expressing B cells expressed higher levels of surface sIgG than the control M2.Stop retrovirus transduced cells. Similarly, the M2 and M2.Stop transduced populations both upregulated CD138, although the presence of M2 did not lead to the high levels of CD138 indicative of plasma cell differentiation. Notably, the other changes observed in B cell differentiation were unique to the M2-transduced B cell population versus the cells transduced with the M2.Stop control retrovirus. In addition, the M2-transduced B cells secreted significantly higher levels of IgG on days 4–6 post-transduction than the cells transduced with the control retrovirus (Figure 3B). Secreted IgM levels remained similar throughout the time-course for M2 and control retrovirus B cell cultures (Figure 3C). Importantly, M2-transduced cells express surface IgG and remain CD138^low^, indicating that they have not fully differentiated into plasma cells. Together, these data provide strong evidence that M2 expression leads to B cell activation and differentiation similar to a functional activated, pre-plasma memory B cell phenotype, namely CD19^+^, sIgG^+^, sIgD^−^, B220^low^, CD138^low^ [33],[34]. However, we cannot formally rule out that M2 expression leads to differential survival and expansion of a population of pre-plasma memory B cells present in the transduced culture – although this seems unlikely based on the very low frequency of this population in the purified naïve splenic B cells used for these studies.

### M2 protein expression leads to secretion of IL-10

**Figure 4:**
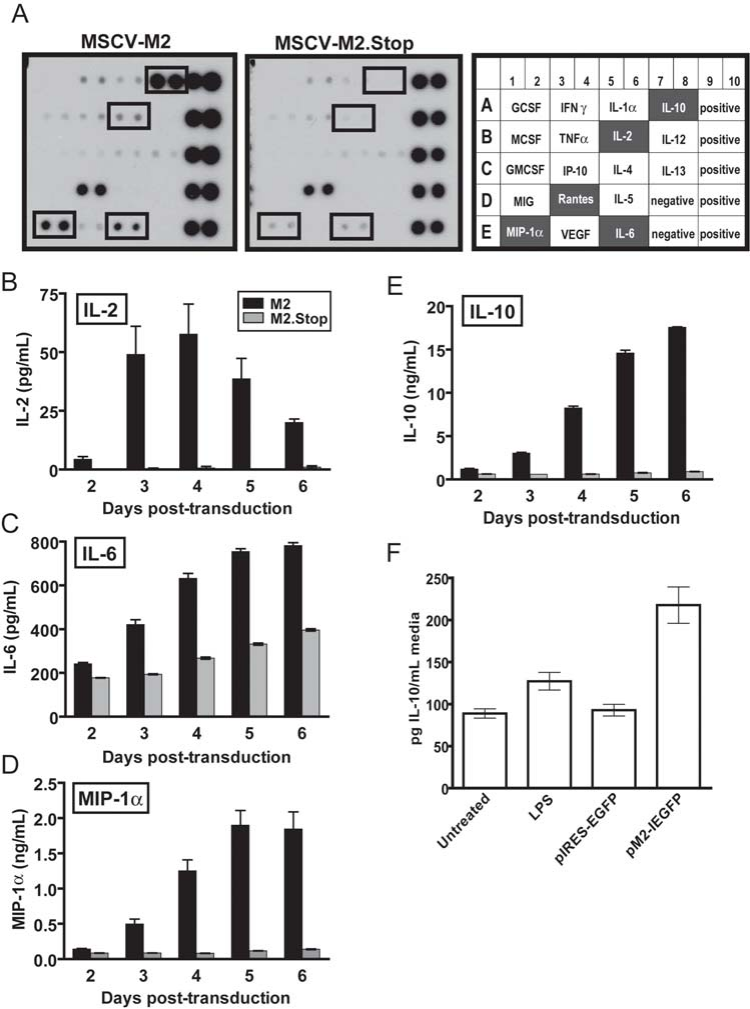
M2 expressing cells secrete more IL-2, IL-6, MIP-1α, and IL-10. (A) Cytokine antibody arrays were screened for the presence of cytokines in the supernatants of M2 and M2.Stop transduced B cell cultures at day 4 post-transduction. Data is representative of supernatants from two independent time-course experiments. (B–E) ELISAs of IL-2, IL-6, MIP-1α, and IL-10 levels in the culture supernatants M2 and M2.Stop transduced B cells. Data shown from triplicate wells analyzed from a single time-course analysis. The data shown are representative of three independent experiments. (F) IL-10 ELISAs of culture supernatants from the murine A20 cell line, either transfected with an empty plasmid (pBluescript II SK) and untreated, treated with LPS, or following transfection with a control expression plasmid (pIRES-EGFP) or an M2 expression plasmid (pM2-IEGFP). The difference between the levels of IL-10 in the culture media of vector control and M2 expression vector transfected A20 cells was statistically significant (p = 0.0054).

To further investigate the proliferative effects of M2 protein expression in primary murine B cells, the supernatants of the transduced B cells were screened for a variety of cytokines using a mouse cytokine antibody array (see Materials and Methods). Supernatants of B cell cultures transduced with M2 and control retrovirus were compared at four days post-transduction (Figure 4A). Cytokine arrays performed in duplicate time-course experiments revealed substantial increases in IL-10, IL-2, IL-6, and MIP-1α in the culture supernatants of B cells expressing M2 compared to the control retrovirus transduced B cell cultures (Figure 4A). Cytokine levels throughout the time-course analyses were subsequently quantitated by ELISA. IL-2 levels in the M2-transduced cultures peaked at 50 pg/mL of supernatant at day 4 and waned by day 6 post-transduction, while only 1–2 pg/mL of IL-2 were detected in the control retroviral supernatants (Figure 4B). From 3 days post-transduction until the end of the time-course, the supernatants from M2-expressing B cells contained levels of IL-6 twice as high as those of B cells transduced with the control retrovirus (780 pg/mL vs. 400 pg/mL) at day 6 post-transduction (Figure 4C). There was also a 10-fold increase in the level of MIP-1α with M2-transduced cultures containing an average of 1845 pg/mL of MIP-1α versus 139 pg/mL in the control retrovirus supernatant at the end of the time course (Figure 4D). Notably, we observed a 20-fold increase in IL-10 levels in the B cell cultures transduced with M2 with 17.5 ng/mL of IL-10 in the M2-transduced cultures as compared to 0.9 ng/mL in the control retroviral supernatants at day 6 post-transduction (Figure 4E). Notably, the number of cells in the M2 protein expressing and control B cell cultures were not significantly different, and thus the observed differences in cytokine levels cannot be explain by an increase in cell number. These data demonstrate that M2 expression in primary murine B cells leads to enhanced secretion of several cytokines, most notably IL-10. Finally, to further assess the ability of M2 expression to up-regulate IL-10 secretion from B cells, we transfected the murine A20 B cell line with either a control expression vector (pIRES-EGFP) or an M2 expression vector (pM2-IEGFP) and assessed IL-10 secretion by ELISA (Figure 4F). Untreated A20 cells secrete significant levels of IL-10, which were only modestly enhanced by LPS treatment (Figure 4F). In addition, transfection of the control expression vector had no impact of the levels of IL-10 secreted by A20 cells (Figure 4F). However, transfection with the M2 expression vector lead to a substantial increase in the levels of IL-10 secretion (Figure 4F). The latter result provides further evidence that M2 is able to increase IL-10 secretion by B cells – independent of LPS stimulation.

### IL-10 is required for the M2-driven B cell proliferation

**Figure 5:**
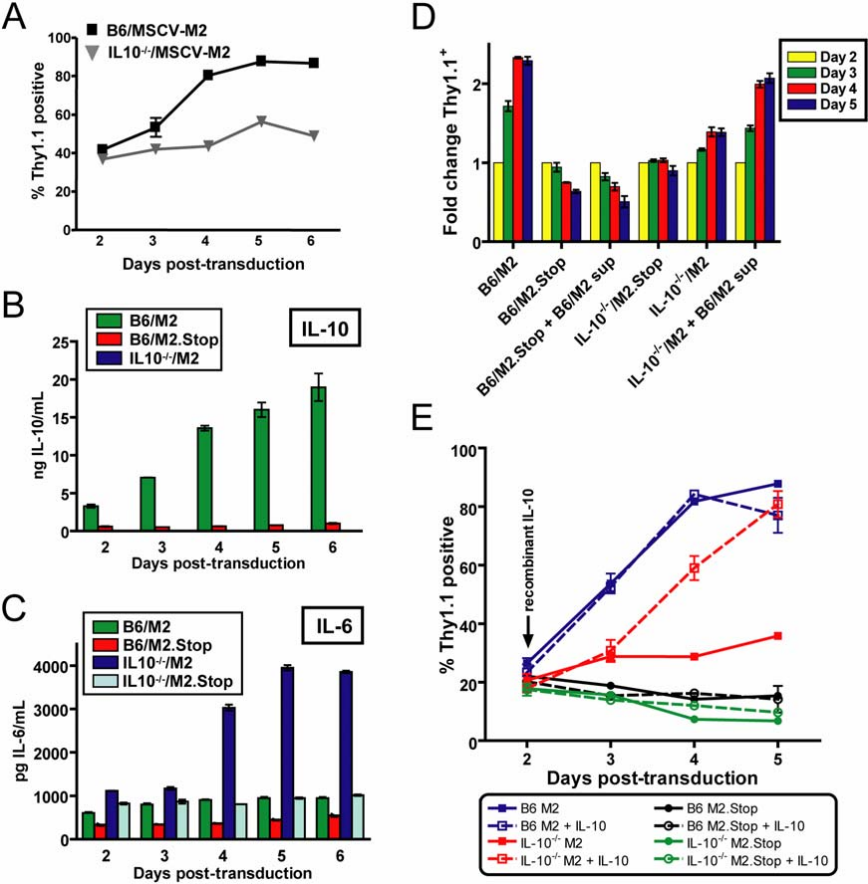
M2 expressing B cells fail to expand in the absence of IL-10. (A) B cells from C57Bl6 and IL-10^−/−^ mice were isolated from splenocytes and transduced with either the M2 or M2.Stop retroviruses. Triplicate wells were analyzed per time-point. IL-10^−/−^ B cells transduced with M2 failed to expand to the same extent as wild-type B cells. Data shown are representative of 3 independent experiments. (B) Quantitative ELISA analyses confirm that IL-10^−/−^ B cells fail to secrete IL-10. (C) IL-10^−/−^ B cells transduced with M2 secrete significantly higher levels of IL-6 than C57Bl/6 B cells transduced with M2. However, IL-6 fails to compensate for the IL-10 deficiency. Data shown is representative of two independent experiments, each containing triplicate cultures. (D) Supernatants from C57Bl6 B cells transduced with M2 compliment for the IL-10^−/−^ B cell proliferation defect. B cells from C57Bl6 and IL-10^−/−^ mice were isolated and triplicate cultures transduced with M2 or M2.Stop retroviruses. On day 2 post-transduction, 500 µL of supernatant from the C57Bl6 B cells transduced with the M2.Stop retrovirus and IL-10^−/−^ B cells transduced with the M2 retrovirus were replaced with 500 µL of tissue culture supernatant recovered from the C57Bl6/M2 B cell cultures from that respective day. Triplicate wells were analyzed per time-point. High IL-10 levels fail to complement the lack of M2 expression in the C57Bl/6 B cells, but did drive proliferation of the M2-transduced IL-10^−/−^ B cells. Data are represented as the fold-change in percent Thy1.1 positive cells in the cultures over the percent Thy1.1 positive cells present at two days post-transduction. (E) Recombinant murine IL-10 rescues M2-mediated expansion of IL-10^−/−^ B cells, but fails to expand either the C57Bl/6 or IL-10^−/−^ M2.Stop transduced B cell populations. B cells from C57Bl6 and IL-10^−/−^ mice were isolated from splenocytes and transduced with either the M2 or M2.Stop retroviruses. IL-10 was added (final concentration of 20 ng/ml) to the indicated samples starting on day 2 post-transduction (see text for description). Triplicate wells were analyzed per time-point.

IL-10 has been demonstrated to be involved in the establishment of a latent MHV68 infection, and we asked whether IL-10 played a role in M2-driven B cell proliferation [35],[36]. To address the role of IL-10 in M2-driven proliferation, B cells were isolated from wild-type and IL-10^−/−^ mice, transduced with M2 or the control retrovirus, and surface Thy1.1^+^ expression was monitored over a six day time course. Although the percentage of M2-transduced C57Bl/6 B cells increased from 40% to 85% of the culture, as previously observed (see Figure 1C), there was only a modest expansion of the Thy 1.1^+^ population from 37% to 49% in the IL-10^−/−^ cultures transduced with M2 expressing MSCV retrovirus (Figure 5A). ELISAs of the supernatants from the transduced cultures confirmed that the IL-10^−/−^ B cells do not secrete detectable levels of IL-10 (Figure 5B). We noted an approximately 10% increase in the percentage of IL-10^−/−^ M2 protein expressing B cells over the time course experiments, and we hypothesize that this small increase might be due to the ability of the M2 protein to manipulate proliferation and/or survival pathways independent of IL-10. Notably, IL-10^−/−^ mice have been shown to have 20-fold higher levels of serum IL-6 than IL-10-sufficient mice [37], and indeed we observed a two-fold increase in IL-6 in the IL-10^−/−^ B cell supernatants of the untransduced population at day 2 (Figure 5C). Expression of the M2 protein led to a four-fold increase in the levels of IL-6 in the culture supernatants of IL-10^−/−^ B cells (Figure 5C). In addition, MIP-1α levels were four-fold higher in the IL-10^−/−^ B cell cultures at day 2 post-transduction, and this increase was observed throughout the time-course (data not shown). However, the increased levels of IL-6 and MIP-1α observed in the M2-transduced IL-10^−/−^ cultures could not compliment the loss of IL-10 in the cultures, leading us to hypothesize that IL-10 secretion is required for the expansion of the M2 protein-expressing B cells.

To more directly assess the role of IL-10 in M2 protein-mediated B cell proliferation and survival, we tested the ability of the cytokine enriched supernatants from transduced wild-type and IL-10^−/−^ B cells to compliment loss of M2 and IL-10 expression in culture. After analysis of transduction efficiency at two days post-transduction, one third of the supernatant from the WT MSCV-M2.Stop and IL-10^−/−^ MSCV-M2 transduced cultures was replaced with an equal volume of supernatant from C57BL6 MSCV-M2 cultures from the respective days post-transduction. B cells were analyzed for Thy1.1 expression for the remainder of the time-course, and IL-10 levels were measured by ELISA (data not shown). Interestingly, addition of culture supernatants from C57BL6 M2 protein expressing B cells to C57BL6 B cells transduced with MSCV-M2.Stop failed to induce significant proliferation of the transduced B cells (Figure 5D). In contrast, addition of IL-10 containing culture supernatants to IL-10^−/−^ B cells expressing the M2 protein led to a steady proliferation nearly equivalent to that of C57BL6 B cell cultures expressing the M2 protein (Figure 5D).

Finally, to formally demonstrate that IL-10 is required for the observed phenotype, we transduced IL10^−/−^ B cells with either the M2 or M2.Stop control recombinant MSCV viruses and assayed the frequency of Thy 1.1. cells in the culture over time in the presence and absence of recombinant IL-10 (Figure 5E). As expected, the addition of recombinant IL-10 had no discernable effect on M2 expressing IL-10-sufficient B cells recovered from C57Bl/6 mice. However, addition of IL-10 to the M2 transduced IL-10^−/−^ B cells (but not the M2.Stop transduced IL-10^−/−^ B cells) rescued the dominance phenotype (Figure 5E). These results demonstrate that both intracellular M2 expression and IL-10 secretion are necessary for the observed proliferative expansion of the transduced B cell population, and that neither one alone is sufficient to induce this expansion. These data suggest that M2 manipulates intracellular signaling pathways which enhance the response to IL-10 signaling as well as induce IL-10 secretion.

### Loss of M2 protein expression during MHV68 infection results in a significant reduction in serum IL-10 levels

**Figure 6:**
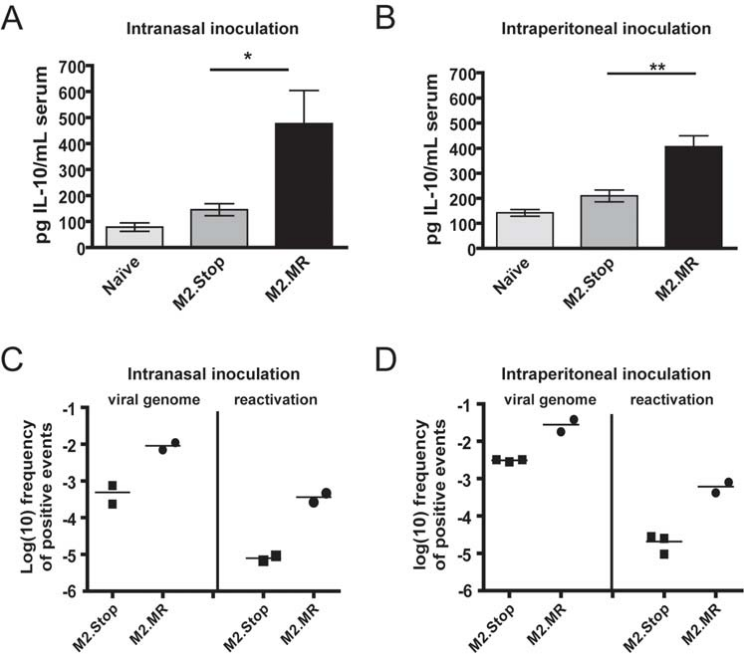
Loss of M2 expression *in vivo* correlates with reduced serum IL-10 at the onset of latency. The reduction in serum IL-10 is independent of the route of infection and correlates with a significant reduction in reactivation from latency in the absence of M2. Groups of four to five mice were infected with 1000 pfu of M2 null mutant (MHV68/M2.Stop) or the M2 marker rescue virus (MHV68/M2.MR) via intranasal inoculation or 100 pfu via intraperitoneal inoculation (or naïve mice). On days 14 or 15 post-infection, mice were injected with biotin labeled anti-IL-10 antibody i.p. Twenty-four hours later, serum was collected for analysis of IL-10 levels (A and B), and splenocytes were recovered for determinations of the frequency of viral latency and reactivation (C and D). Data shown for intranasal inoculations is representative of two independent experiments, 4–5 mice per group, with IL-10 levels measured in individual mice and splenocytes pooled for viral latency assays (as described in Materials and Methods). Data shown for intraperitoneal inoculations is representative of three independent experiments, 4–5 mice per group, with IL-10 levels measured in individual mice and splenocytes pooled for viral latency assays. (A and B) Total serum IL-10 levels were significantly lower in mice infected with MHV68/M2.Stop compared to MHV68/M2.MR virus, independent of the route of infection. Intranasal infection data is from 10 individual mice from two independent infections; intraperitoneal data is from 15 individual mice from three independent infections. Two naïve animals were analyzed per infection * p = 0.0117, ** p = 0.0005. Significance of IL-10 data was determined by two-tailed, unpaired Student's T test with a confidence level of 95%. (C and D) Frequencies of splenocytes from MHV68/M2.MR and MHV68/M2.Stop infected mice harboring latent viral genomes and reactivating from latency upon explant. The frequency of latency was determined by nested, limiting-dilution PCR (LD-PCR) as previously described [3]. The frequency of cells reactivating from latency upon in vitro culture was determined by plating serial dilutions of live, intact splenocytes on mouse embryonic fibroblast monolayers and scoring cytopathic effect 14–21 days post-explant, as previously described [3]. Data from both assays was subjected to nonlinear regression analysis with a sigmoidal dose-response algorithm for best fit. Data points represent individual experiments with splenocytes pooled from 4–5 mice per condition.

Previous studies have shown that in the absence of a functional M2 gene, establishment of MHV68 latency following intranasal inoculation is severely reduced [24]. Similarly, inoculation of IL-10^−/−^ mice with wild-type MHV68 leads to a decrease in the establishment of latency [35],[36]. To determine whether M2 expression leads to IL-10 secretion *in vivo*, C57Bl/6 mice were infected (either 1,000 pfu via intranasal inoculation or 100 pfu via intraperitoneal inoculation) with either a recombinant MHV68 harboring the same translation termination codon near the 5′ end of the M2 open reading frame as used in control retrovirus construction (MHV68/M2.Stop) or with a genetically repaired marker rescue isolate of the same locus (MHV68/M2.MR). Both intranasal and intraperitoneal inoculation of the M2-null mutant were assessed, since we have previously reported that route of inoculation impacts the latency phenotype observed [24]. Serum IL-10 was measured by *in vivo* cytokine capture and ELISA (Figure 6, panels A & B). Notably, mice infected with MHV68/M2.Stop had serum IL-10 levels that were only slightly elevated over the levels present in naïve mice and were 2- to 3-fold lower than the levels observed in mice infected with the marker rescue virus (MHV68/M2.MR). Notably, this phenotype was independent of the route of inoculation (Figure 6, panels A & B). As we have previously reported [24], we observed defects in both establishment of latency (which was accentuated following intranasal inoculation), as well as reactivation from latency with the M2-null mutant MHV68 (Figure 6, panels C & D). Intraperitoneal infection with MHV68/M2.Stop increased the establishment of latency eight-fold over intranasal inoculation, yet serum IL-10 levels were very similar to those observed following intranasal inoculation (Figure 6). Importantly, the serum levels of IL-10 we observed in MHV68/M2.MR infected mice were similar to those previously observed [35]. These results provide strong evidence that M2 induction of IL-10 secretion, either from latently infected B cells or some other latency reservoir (e.g., infected macrophages or dendritic cells), contributes significantly to the serum levels of IL-10 observed during MHV68 infection following either intranasal or intraperitoneal virus inoculation.

### Loss of M2 expression during MHV68 infection correlates with an increase in virus-specific, activated CD8^+^ T cells

**Figure 7:**
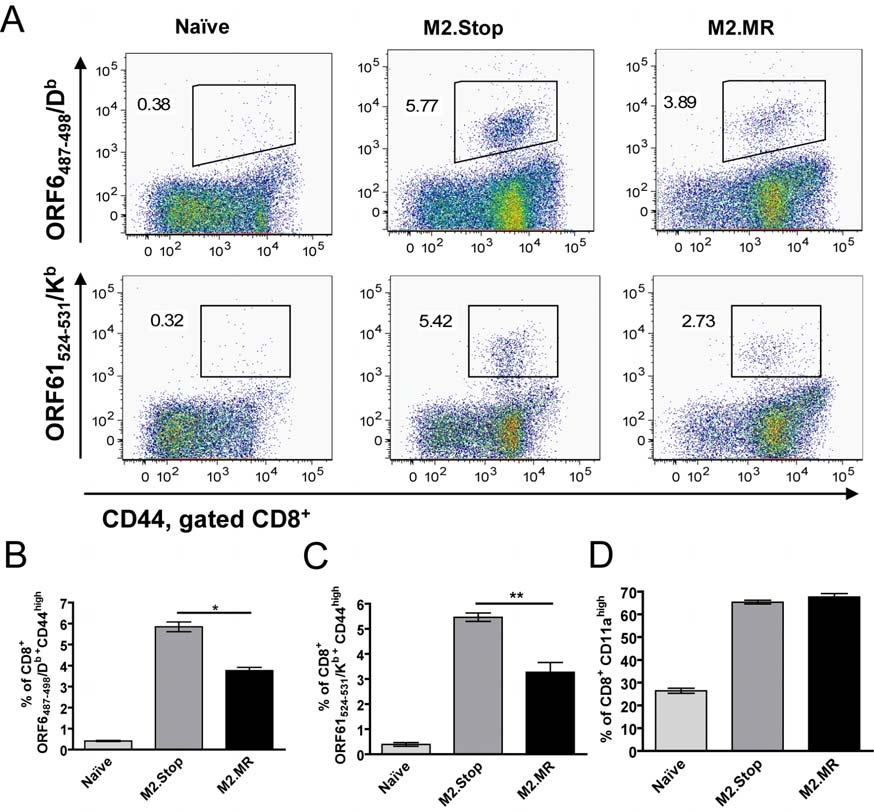
Loss of M2 protein expression and reduction of serum IL-10 levels correlates with an enhancement in the MHV68-specific tetramer response. Groups of four to five mice were infected with 100 pfu via intraperitoneal inoculation with either MHV68/M2.Stop or MHV68/M2.MR. Sixteen days post-infection, at the onset of latency, splenocytes were analyzed by flow cytometry. (A) Splenocytes were stained with tetramers for two MHV68 lytic antigens, ORF6_487-498_/D^b^-APC or ORF61_524-531_/K^b^-APC, anti-CD8-PacificBlue, and anti-CD44-FITC. The data shown is from mice with an intermediate phenotype representative of the median in each group. (B and C) Mean percentage of ORF6-specific (panel B) and ORF61-specific (panel C) T cells was significantly higher in the absence of the M2 protein (* p<0.0001, ** p = 0.0009). Tetramer data was compiled from the analysis of individual mice and represents two independent infections with 5 mice per group per infection. (D) Mean percentage of activated CD8^+^ T cells did not significantly change in the absence of the M2 protein. Data shown is from one representative set of infections of five mice from three independent experiments. Statistical significance of the flow cytometry data was determined by two-tailed, unpaired Student's T test with a confidence level of 95%.

We next examined whether loss of M2 expression and the concomitant reduction in IL-10 expression might alter the CD8 T cell response to MHV68 since IL-10 is known to suppress T cell responses [18]. Thus, we examined the MHV68-specific CD8^+^ T cell response following infection of mice with either MHV68/M2.Stop or MHV68/M2.MR. Mice were infected intraperitoneally with 100 pfu of MHV68/M2.Stop or MHV68/M2.MR and splenocytes were harvested at day 16 post-infection, a time at which lytic virus has been cleared and latency established. As previously reported, there was a ten-fold decrease in establishment of latency with a 20-fold decrease in reactivation (Figure 6D). Splenocytes from individual mice were stained for activated, tetramer positive CD8^+^ T cells using tetramers specific to two MHV68 antigens encoded by ORF6 and ORF61 (Figures 7, panels A–C). Both tetramers used in this analysis were specific for viral antigens expressed during the virus lytic replication cycle. In two independent experiments, tetramer staining for two different lytic antigens revealed a statistically significant increase in the frequency of tetramer-specific, activated CD8^+^ T cells in mice infected with MHV68/M2.Stop compared to MHV68/M2.MR (Figure 7A–C). In contrast, there was no global change in overall CD8 activation as determined by the percentage of CD8^+^ CD11a^high^ T cells in the spleens of infected mice (Figure 7D). CD4^+^ T cell activation, as well as the percentage of CD44^high^ CD62L^low^ CD4^+^ and CD8^+^ T cells, was the same in the two groups of infected mice (data not shown). These data indicate that the loss of M2 during MHV68 infection specifically enhanced the MHV68-specific CD8^+^ T cell response, despite a significant decrease in viral latency and reactivation (see Figure 6D).

Overall, the immune response in the absence of M2 protein expression during infection is unique in that the MHV68-specific T cell response is increased correlating with a decrease in serum IL-10 levels. These data point to a potential role of M2 protein-mediated IL-10 secretion in the quiescence of the virus-specific T cell response *in vivo* which may facilitate both the efficient establishment of latency as well as reactivation from latency.

## Discussion

The latency-associated M2 protein is critical for establishing splenic latency following low dose intranasal inoculation and for virus reactivation from latency following low dose intraperitoneal inoculation [23],[24]. In the absence of M2, infected B cells are unable to efficiently transition from the germinal center to the follicles [26]. Early in latency, there is an accumulation of latently infected naïve B cells in the absence of the M2 protein, indicating a role for the M2 protein in manipulating B cell development during infection [24]. Epstein-Barr virus is hypothesized to drive naïve B cells to enter the germinal center reaction in order to establish latency in the memory B cell pool [7],[38],[39]. In long-term EBV carriers, lytic EBV gene transcripts are preferentially found in the plasma cell population, leading to a model whereby reactivation from latency is associated with differentiation from memory to plasma cell [40]. B cell proliferation is necessary for the establishment of MHV68 latency, and, similar to EBV, memory B cells are the primary long-term latency reservoir [8],[9],[30]. Reactivation is hypothesized to be needed for efficient seeding of the spleen during the establishment phase of MHV68 infection, and, as such, the M2-associated defects in establishment of latency and reactivation from latency may, in fact, be functionally linked. In this study we explored the impact of M2 protein expression in primary murine B cells - a system capable of differentiation.

M2 expression in primary B cells led to proliferation of transduced B cells, driving a rapid expansion of transduced cells within the culture, regardless of initial transduction efficiency. Although both the transduced (Thy1.1^+^) and untransduced (Thy1.1^−^) B cell populations could be shown to be proliferating (by BrdU incorporation), the enhanced proliferation and survival of the M2-transduced B cells rapidly led to this population dominating the mixed culture. In primary murine B cells, M2-driven proliferation was dependent on the B cell's ability to secrete IL-10 and respond to IL-10 signaling. Notably, transfer of culture supernatants from M2 expressing C57Bl/6 B cells, or addition of recombinant murine IL-10, did not result in dominance of the M2.Stop retrovirus transduced (i.e., Thy1.1^+^) population in the absence of M2 protein expression, leading us to hypothesize that some other function(s) of the M2 protein augments IL-10 signaling. Culturing stimulated human memory B cells with IL-10 or IL-2 and IL-6 leads to plasma cell differentiation [41],[42]. Also, an increase in MIP-1α transcription is associated with differentiation to a plasma cell phenotype [43]. Human germinal center B cells can be induced to differentiate into plasma cells rather than memory B cells in the presence of IL-10 [44]. In contrast to human B cells, IL-10 enhances murine B cell viability but does not drive proliferation [19]. Our data suggest that the MHV68 M2 protein uniquely increases the murine B cell proliferative response to IL-10, mimicking the role of IL-10 signaling in human B cells.

M2-transduced B cells were B220^low^, I-A^b low^, sIgD^−^, yet retained surface expression of CD19 and IgG, and remained CD138^low^, indicating that they did not fully differentiated into plasma cells. Instead, the surface phenotype of the B cells expressing M2 most closely resembled that of a pre-plasma memory B cell, an intermediate stage in development between the memory and plasma cell phenotypes [33],[45]. Together, this data supports a model wherein infection of naïve B cells in the lung with MHV68 leads to M2 expression, B cell proliferation and activation, and differentiation to a pre-plasma memory B cell phenotype. Depending on other cytokines and signals in the area, M2-expressing B cells may further differentiate into memory B cells, establishing long-term latency, or plasma B cells, potentiating virus reactivation. Thus, in this model of MHV68 pathogenesis, M2 protein manipulation of B cell differentiation to an intermediate pre-plasma memory B cell phenotype could facilitate both virus reactivation as well as establishment of viral latency.

IL-10 has potent immunoregulatory activity, suppressing proinflammatory cytokine secretion and activation of antigen-presenting cells - functions which result in suppressed NK cell and T cell activity [46]. IL-10 plays an important role in MHV68 pathogenesis, but prior to our analyses of M2 protein function no specific viral antigen had previously been shown to stimulate cellular IL-10 production. It has been shown that ex vivo stimulation of MHV68 latently infected splenocytes with MHV68-infected antigen presenting cells resulted in IL-10 secretion peaking at the onset of splenic latency, and B cells were shown to be responsible for a significant portion of the IL-10 secreted [47]. Dendritic cells isolated from MHV68 infected mice express IL-10 transcripts, and dendritic cells infected ex vivo secrete IL-10 only when concurrently stimulated with LPS [36]. Interestingly, these investigators showed that M2 is transcribed by infected dendritic cells, although they did not demonstrate that IL-10 secretion was mediated by M2 [36]. In the absence of IL-10, establishment of MHV68 latency is decreased concurrent with an increase in serum IL-12 p70 and splenomegaly, demonstrating a role for IL-10 in both establishment of latency as well as immunosuppression [1],[35],[47].

We observed a significant decrease in serum IL-10 in mice infected with an M2-null MHV68 mutant. Notably, the decreased serum IL-10 levels correlated with an increase in the percentage of MHV68-specific CD8^+^ T cells. Furthermore, it is important to note that this increased CD8+ T cell response was in the setting of an infection where virus reactivation was severely attenuated. Therefore, increased persistent virus replication cannot explain the increase in the tetramer-specific response. Thus, we hypothesize that during M2-mediated reactivation there is concurrent IL-10 secretion, locally dampening the ability of the MHV68-specific CD8^+^ T cells to clear the infected cells, leading to enhanced establishment and reactivation from latency.

Manipulation of the IL-10 signaling pathway appears to be a conserved mechanism used by a number of herpesviruses. EBV encodes a viral IL-10 homolog, BCRF1 (or vIL-10), that has been shown to increase human B cell proliferation following surface immunoglobulin crosslinkinking and induce B cells to secrete increased levels of IgM, IgG, and IgA in a similar manner to cellular IL-10 [17]. In the absence of vIL-10, EBV can still efficiently establish latent, long-term lymphoblastoid lines (LCLs) [48]. Exogenous vIL-10 added during the transformation of B cells by EBV enhanced both the rate and frequency of growth transformation, and antisense oligonucleotides to vIL-10 could negate this enhancement [49],[50]. Human IL-10 could complement the loss of vIL-10 during EBV infection of B cells, demonstrating that it is IL-10 mediated signaling that augments B cell transformation following EBV infection [50]. LMP1, a functional CD40 ortholog encoded by EBV, is both IL-10 responsive and induces secretion of cellular IL-10 in stimulated Burkitt's lymphoma cells [51],[52]. Patients with EBV-associated post-transplant lymphoproliferative disease also have elevated serum IL-10, but not IL-6 [53]. However, *in vivo*, whether the primary role of vIL-10 is to suppress the immune response, trigger B cell proliferation and differentiation or both is unclear. Serum cellular IL-10 levels are elevated both during primary EBV infection as well as during EBV reactivation from latency, implying that IL-10 plays a role in both the establishment and reactivation from latency [54].

Human cytomegalovirus (HCMV), a beta herpesvirus, encodes a viral IL-10 (cmvIL-10) that has only 27% homology to cellular IL-10, but is nevertheless capable of binding the IL-10 receptor and mediating downstream STAT1/STAT3 signaling [55]. Human cmvIL-10 is capable of downregulating MHC I and II, suppressing PBMC proliferation, and decreasing IFNγ, IL-1α, GM-CSF, IL-6, and TNF-α secretion in response to stimulation [56]. Transcripts encoding cmvIL-10 have been detected in the bone marrow and mobilized peripheral blood during natural HCMV latency, indicating that cmvIL-10 may play a role in either establishment, maintenance, or reactivation from latency [57]. Murine CMV (MCMV) does not encode an IL-10 homolog, although, parallel to the studies of Flano et al. on dendritic cells infected with MHV68 [36] , *in vitro* MCMV infection of primary macrophages results in secretion of cellular IL-10 and downregulation of MHC II [58]. Recently, IL-10 production by CD4 T cells has been shown to be of key importance in regulating MCMV persistence in the salivary glands. Blockade of the IL-10R resulted in a significant decrease in the titer of MCMV in the salivary glands with a concurrent increase in the frequency of IFNγ-producing CD4 T cells, indicating a role for IL-10 in the maintenance of a MCMV infection, possibly through reactivation from latency [59]. It seems reasonable to speculate that suppression of the host response may help these viruses both establish latency as well as reactivate from latency, reseeding the latency reservoir without clearance by memory T cell responses.

IL-10 has been implicated in the pathogenesis of both autoimmune as well as viral diseases, and the fact that many viruses carry IL-10 orthologs speaks to the potency of IL-10 in manipulating the host immune system [60],[61]. The parapoxvirus, ORF virus, encodes a viral IL-10 homolog with 80% homology to ovine IL-10 and is capable of inhibiting T cell proliferation [62]. Finally, two independent reports have demonstrated that blockade of the IL-10 receptor during chronic lymphocytic choriomeningitis virus infection led to clearance of the infection with enhanced IL-10 production by dendritic cells [63],[64].

We also observed a significant increase in IL-6 and MIP-1α in the B cell cultures transduced with M2. Interestingly, KSHV encodes homologs of both IL-6 and MIP-1α, suggesting that these cytokines have key roles in gammaherpesvirus immunomodulation that we have yet to appreciate [16]. MIP-1α can be detected in the BAL and lung homogenate of MHV68-infected mice at the peak of lytic replication, but the contribution of this cytokine to latency and reactivation has not been studied directly [65],[66]. During MHV68 infection, MIP-1α secretion in the lungs may attract B cells to the area of acute replication, facilitating viral infection and trafficking to the spleen. There is no significant difference in pathology or viral latency in IL-6^−/−^ mice, despite the fact that upon ex vivo stimulation infected splenocytes secrete IL-6 [67]. Further study is necessary to explore the links between M2 and these cytokines.

Does M2-driven IL-10 secretion play a critical role in MHV68 latency? The studies presented here provide an indication that the M2 protein has multiple functions – some of which are necessary for primary murine B cells to respond to IL-10 signaling in the retroviral transduction assays we have described. Our attempts to neutralize IL-10 in the B cell culture system have been unsatisfactory (data not shown) – perhaps owing to the difficulty of neutralizing the autocrine activity of IL-10 expressed from primary murine B cells. Thus, we anticipate that attempting to neutralize IL-10 in vivo during MHV68 infection will be difficult. As a distinct approach, we have recently published the analysis of a panel of point mutations in candidate functional motifs in the M2 protein [68]. The latter studies have also provided evidence for the presence of multiple functionally important domains in M2 [68]. With respect to M2-driven B cell proliferation and IL-10 secretion, we analyzed in primary B cells three M2 mutants which, in the context of virus infection, were severely attenuated in establishment and reactivation from MHV68 latency. Notably, two of these mutations (Y129F/P7 and P8) ablated the IL-10 dependent proliferative dominance phenotype in primary B cell cultures while the other mutation (P9) was similar to wild type M2 [68]. The latter result underscores that M2 is a multifunctional protein. In addition, these studies link M2 functional domains that play a critical role in MHV68 latency in vivo to M2-driven IL-10 secretion. Further studies will be required to assess the contribution of M2-driven IL-10 expression to chronic MHV68 infection.

In summary, the analysis of M2 protein function provides a unique insight into an immunomodulatory mechanism that is employed by many viruses, particularly the herpesvirus family. Our work demonstrates that the M2 protein, a unique viral protein, manipulates B cell signaling to induce cellular IL-10 secretion and make cells more responsive to IL-10 signaling, leading to proliferation and enhanced survival of M2-expressing primary B cells in culture. M2 expression in primary murine B cells results in differentiation to a pre-plasma memory B cell phenotype, an intermediate in mature B cell development. In addition, M2 protein expression correlates with high serum IL-10 levels and an increased frequency of virus-specific CD8^+^ T cells during MHV68 infection. We conclude that driving B cell proliferation, survival and differentiation, while simultaneously dampening the host immune response to the virus, is an elegant immunomodulatory mechanism used by MHV68 to both facilitate establishment of latency and subsequent episodic virus reactivation from latency.

## Methods

### Mice and infections

Female C57Bl/6 and IL-10^−/−^ mice 6 to 8 weeks of age were purchased from the Jackson Laboratory. Mice were sterile housed and treated according to the guidelines at Emory University School of Medicine (Atlanta, GA). Following sedation, mice were infected intranasally with 1000 pfu of either MHV68/M2.Stop or MHV68/M2.MR in 20 µL of cMEM. Mice were infected with 100 pfu of MHV68/M2.Stop or MHV68/M2.MR in 500 µL of cMEM intraperitoneally. Mice were allowed to recover from anesthesia before being returned to their cages.

### B cell isolation

Spleens were homogenized and erythrocytes removed by hypotonic lysis. B cells were enriched using negative selection by magnetic cell separation with the mouse B Cell Isolation Kit (Miltenyi Biotech). Purity was confirmed by staining for CD19, and B cells used in experiments were 93–97% pure. Cells were cultured in RPMI-1640 supplemented with 10% FCS, 100 U/mL penicillin, 100 mg/mL streptomycin, 2 mM L-glutamine, 10 mM HEPES, 1 mM sodium pyruvate, 10 mM non-essential amino acids, and 25 µg/mL of LPS (Sigma) overnight before retroviral transduction.

### Cloning, retroviral production, and transduction

BglII sites were cloned flanking the M2 ORF with primers 5′ CAG CTC AGA TCT ATG GCC CCA ACA CCC 3′ and 5′ CAG CTC AGA TCT TTA CTC CTC GCC CCA 3′ and cloned into pCR-Blunt (Invitrogen). Positive clones were sequenced, digested with BglII, and cloned into the pMSCV-IRES-Thy1.1 vector (a gift from Philippa Marrack) to construct pMSCV-M2-IRES-Thy1.1. pMSCV-M2Stop-IRES-Thy1.1 was constructed in a similar manner. Retroviruses were produced using the BOSC23 producer cell line (ATCC). 2×10^6^ BOSC23 cells were plated on 60 mM Collagen II coated plates. The following day, 10 ug of pMSCV vector was transfected into the BOSC23 cells using the LT-293T reagent from Mirus Biotech. Retroviral supernatants were harvested 48 to 72 hours post-transfection, centrifuged at 2000 rpm for 10 minutes to clear cell debris, and supplemented with 5 µg/mL of polybrene. B cells were transduced by removing 700 µL of media and replacing it with 1 mL of retroviral supernatant/polybrene. Cells were spun at 2500 rpm at 30°C for one hour. 750 uL of retroviral supernatant was removed and replaced with fresh, complete RPMI. Cells were rested for 48 hours before analysis.

In some analyses recombinant IL-10 was added back to transduced primary B cell cultures, as follows. Primary murine B cells were harvested from C57Bl6 and IL-10^−/−^ mice as previously described. Transduction efficiencies were measured 48 hours post-transduction by flow cytometry. Following day 2 analysis, indicated cultures received 20 ng/mL of murine recombinant IL-10 (Peprotech). B cell populations were analyzed on days 3–5 post-transduction by flow cytometry.

### Immunoprecipitations and western blotting

Cells were lysed in a suitable volume of ELB buffer on ice for 20 minutes. Lysates were pre-cleared with pre-immune chicken IgY, and M2 precipitated with chicken anti-M2 IgY followed by capture by agarose anti-IgY beads (Aves Labs, Inc.). Precipitates were run on a 15% acrylamide gel, transferred to nitrocellulose membranes, and blotted with rabbit anti-M2 antisera followed by donkey anti-rabbit HRP. Protein was detected using chemiluminescence on Kodak X-Omat Blue XB-1 film.

### Flow cytometry

Rat anti-mouse CD16/32 (Fc block) was used prior to staining in most experiments. Cells were stained with the following antibodies: Thy1.1-FITC, -PE, or –APC (eBiosciences), CD44-FITC (Caltag), CD62L-PE (Caltag), GL7-FITC, IgG_1, 2a, 2b, 3_-FITC, CD25-PE, CD138-PE, I-A^b^-PE, CD4-PerCP, CD11a-PE-Cy7, CD19-APC, B220-APC, CD8-PacficBlue (BD Pharmigen except where noted). Tetramers were synthesized at the NIH Tetramer Core Facility at Emory University and conjugated to streptavidin-APC (Molecular Probes) according to core protocol. Intracellular bromodeoxyuradine incorporation was measured using BrdU-APC according to the manufacturer's protocol (BD Pharmigen). AnnexinV-PacificBlue and 7-AAD reagents were purchased in the Vybrant® Apoptosis Assay Kit #14 (V35124) from Molecular Probes and used per manufacturer's protocol. Cells were analyzed on FACScalibur or LSR II flow cytometer. Data was analyzed using FlowJo software (TreeStar, Inc., San Carols, CA).

### Cytokine array and enzyme-linked immunosorbent assays (ELISAs)

TranSignal^TM^ Mouse Cytokine Antibody Arrays 1.0 (Panomics, Inc.) were used to screen for secreted cytokines as per manufacturer's instructions. Membranes were blocked in Blocking Buffer for two hours, washed, and then incubated for two hours at room temperature with day four supernatants from B cells transduced with MSCV-M2 or MSCV-M2.Stop. Membranes were washed and incubated with Biotin Conjugated Anti-Cytokine Mix as per protocol. Membranes were washed and incubated with Streptavidin-HRP. After a final wash, bound cytokine was detected using chemiluminescence on Kodak X-Omat Blue XB-1 film. Cytokines were quantitated by ELISA. IL-6 and IL-10 were detected with reagents from BD Biosciences, and IL-2 and MIP-1α were detected with reagents from R&D Biosystems. IgM and IgG were detected with reagents from Bethyl Biosciences.

### Transfection of murine A20 cell line

Triplicate cultures of 1×10^6^ A20 B cells were nucleofected (Amaxa Biosystems) with 4 ug of pIRES2-EGFP (BD Biosciences Clontech), pM2-IRES-EGFP [68], or pBluescriptIISK (Stratagene) using Solution T with setting T-01 on an Amaxa Nucleofector I (Amaxa Biosystems). Cells transduced with pBluescriptIISK were stimulated with 100 ng/mL of LPS following nucleofection as indicated. 48 hours post-nucleofection, supernatants were harvested and secreted IL-10 measured by ELISA (BD Biosciences).

### MHV68 viral mutagenesis

A MHV68 genomic fragment containing the region from bp 2403 to bp 6262 (WUMS sequence) [69] was cloned into the Litmus-38 plasmid (Lit38-M2) as previously described [23]. With Lit38-M2 as a template, a stop codon was introduced into the M2 ORF using the following oligonucleotides: Oligo1 (5′ CCA CCA GGC CGA AGC TTA CGG ATT GGG AAT C) and Oligo2 (5′ CCA ATC CGT AAG CTT CGG CCT GGT GGA TG) generating a translational stop codon at bp 4566 and introducing a *Hind* III restriction site. The resultant product was ligated into the pCR Blunt plasmid (Invitrogen). In addition, an M2 marker rescue pCR Blunt plasmid was generated by PCR using Lit38-M2 as a template and designated as M2.MR. M2.Stop pCR Blunt plasmid and M2.MR were sequenced to verify the introduction of the site directed point mutations and the absence of unwanted mutations. Recombinant viruses were generated by allelic exchange in *E. coli*, as described by Smith and Enquist [70],[71]. Briefly, the *Not* I and *Bam* HI restriction sites within pCR Blunt were used to liberate the MHV68 genomic region contained within the plasmid. This fragment was cloned into the suicide vector pGS284 which harbors an ampicillin gene and a levansucrase cassette for positive and negative selection, respectively. The resulting plasmid was transformed into S17λpir *E. coli* cells and mated to GS500 *E. coli* (RecA^+^) containing wt MHV68 BAC. Cointegrants were selected on Luria-Bertani (LB) agar plates containing chloramphenicol (Cam) and ampicillin (Amp) and were resolved following overnight growth in LB medium with Cam. Next, bacteria were plated on LB agar plates containing Cam and 7% sucrose to select for loss of pGS284 vector sequence. Individual colonies harboring site specific point mutations within M2 were identified by colony PCR followed by restriction digest. Positive clones were grown in LB medium with Cam, and BAC DNA was purified with a Midi Prep Kit (Qiagen, Hilden, Germany) as described by the modified manufacturer's protocol. The presence of site specific point mutations and the absence of unwanted mutations within the region of homologous recombination were confirmed by sequencing and southern blot. Virus stocks were generated by Superfect (Qiagen, Hilden, Germany) transfection of recombinant MHV68 BAC DNA into Vero-Cre cells as previously described [70]. In wells showing cytopathic effect (CPE), virus was harvested, cleared of cell debris, and used to infect Vero-Cre cells in order to generate high-titer stocks. Following the presence of CPE in Vero-Cre cells, samples were harvested, homogenized, clarified, and aliquoted for storage at −80°C. Virus stock titers were determined by plaque assay as previously described [23],[72].

### MHV68 viral assays

Limiting dilution assays for frequency of latent were performed as previously described [23],[24]. To determine the frequency of cells harboring latent viral genomes, single-copy-sensitive nested PCR was performed. Frozen samples were thawed, washed in isotonic buffer, counted, and plated in three-fold serial dilutions in a background of 10^4^ NIH 3T12 cells in 96 well plates. Cells were lysed by protease K digestion for six hours at 56°C. Two rounds of nested PCR were performed per sample with twelve samples per dilution, and the products were resolved on 2% agarose gels. In order to measure the frequency of reactivating splenocytes, bulk splenocytes were resuspended in cMEM and plated in serial two-fold dilutions on mouse embryonic fibroblast (MEF) monolayers in 96-well tissue culture plates. Parallel samples of mechanically disrupted cells were plated to detect preformed infectious virus. Wells were scored for cytopathic effect 14 to 21 days post-explant.

### *In vivo* cytokine capture assay

The Mouse IL-10 *In Vivo* Capture Assay Set (BD Biosciences) was used to detect IL-10 *in vivo* during infections. On day 14–15 p.i., parallel groups of five mice were injected with 10 µg of biotinylated rat anti-mouse IL-10 antibody in 200 µl of sterile PBS. Mice were bled on day 15–16 p.i. and serum collected. Samples were prepared and assayed by ELISA as per protocol. The limit of detection of this assay is 31.3 pg IL-10/mL of serum.

### Statistical analysis

Data analysis was conducted using GraphPad Prism software. Error bars in all graphs depict standard error of the mean. For limiting-dilution analysis, data was subjected to nonlinear regression analysis with a sigmoidal dose-response algorithm for best-fit. Poisson distribution predicts that the frequency at which 63.2% of wells are positive for an event (PCR or reactivation) is the frequency at which there is at least one event present in the population. Statistical significance of the flow cytometry and ELISA data was determined by two-tailed, unpaired Student's T test with a confidence level of 95%.
